# Retraction Note: Preclinical studies of toxicity and anti-cholangiocarcinoma activity of the standardized capsule formulation of atractylodes lancea (Thunb.) DC

**DOI:** 10.1186/s12906-026-05308-3

**Published:** 2026-02-19

**Authors:** Tullayakorn Plengsuriyakarn, Kanawut Kotawong, Juntra Karbwang, Kesara Na-Bangchang

**Affiliations:** 1 Center of Excellence in Molecular Biology and Pharmacology of Malaria and Cholangiocarcinoma, Thammasat University, Pathum Thani, 12120 Thailand; 2 Graduate Program in Bioclinical Sciences, Chulabhorn International College of Medicine, Thammasat University, Pathum Thani, 12120 Thailand; 3 Drug Discovery and Development Center, Thammasat University, Pathum Thani, 12120 Thailand

**Retraction Note: BMC Complementary Medicine and Therapie s23, 186 (2023)**

**https://doi.org/10.1186/s12906-023-03992-z**

The Editor has retracted this article. After publication, concerns were raised regarding highly similar images within the article. Specifically:

• Fig. 1, Thymus (1000) and Fig. 2 Thymus (1000) appear to be highly similar.

• Fig. 1, Brain (1000) and Fig. 2 Brain (1000) appear to be highly similar.

• Fig. 4A, CMC-AL (medium dose) and CMC-AL (low dose) appear to partially overlap.

The authors have stated that the images were included by error but were unable to provide a sufficient response to the concerns raised, nor verifiable original images. Therefore, the Editor no longer has confidence in the presented data.

Tullayakorn Plengsuriyakarn, Kanawut Kotawong and Kesara Na-Bangchang disagree with this retraction.

Juntra Karbwang did not respond to correspondence from the Publisher about this retraction notice.

